# Telescope array bursts, radio pulses and axion quark nuggets

**DOI:** 10.1140/epjc/s10052-022-10208-0

**Published:** 2022-03-22

**Authors:** Xunyu Liang, Ariel Zhitnitsky

**Affiliations:** Department of Physics and Astronomy, University of British Columbia, Vancouver, BC V6T 1Z1 Canada

## Abstract

Telescope array (TA) experiment has recorded Abbasi et al. (Phys Lett A 381(32):2565–2572, 10.1016/j.physleta.2017.06.022, 2017), Okuda (J Phys Conf Ser 1181:012067, 10.1088/1742-6596/1181/1/012067, 2019) several short time bursts of air shower like events. These bursts are very distinct from conventional single showers, and are found to be strongly correlated with lightnings. In our previous work Zhitnitsky (J Phys G 48(6):065201, 10.1088/1361-6471/abd457, arXiv:2008.04325 [hep-ph], 2021) we proposed that these bursts represent the direct manifestation of the dark matter (DM) annihilation events within the so-called axion quark nugget (AQN) model. In the present work we suggest to test this proposal to search for the radio signals in frequency band $\nu \in$ (0.5–200) MHz which must be synchronized with the TA bursts. We argued that the conventional lightning-induced radio emission can be easily discriminated from the AQN-induced radio pulses discussed in this work.

## Introduction

This work is tightly linked to our recent proposal [3] interpreting the mysterious bursts observed by telescope array (TA) experiment [1, 2] in terms of the AQN annihilation events under the thunderstorm. These events are very unusual and cannot be interpreted in terms of conventional single showers as reviewed below. In the present work we shall argue that the proposed mechanism [3] in terms of the AQN annihilation events inevitably predict the radio wave pulses which must be synchronized with TA bursts. Based on this prediction we suggest to test our proposal by searching for the radio signals in frequency band $\nu \in$ (0.5–200) MHz which must be synchronized with the TA bursts, which represents the main goal of the present work.

Such test would unambiguously support or refute the proposal (interpreting TA bursts as the AQN events) because the main arguments of the present work based on synchronization which represents pure geometrical property of the system. This is because the radio pulse and the TA burst are originated from the same location, emitted at the same instant, and propagate with the speed of light. This feature of synchronization is not sensitive to many assumptions and uncertainties which were inevitably present in estimates [3] interpreting the TA bursts as the AQN annihilation events. Furthermore, we also argue that the conventional lightning-induced radio emission is qualitatively different from the AQN -induced radio pulses discussed in this work. Therefore, these two different radio signals can be easily distinguished from each other, and we advocate to conduct such studies.

Finally, before we proceed one should emphasize from the start that AQN events are generically random events as they are related to DM particles. However, as mentioned in [3] the AQN hitting the thunderstorm area may serve as a trigger which sparks the lightning. Therefore, the AQNs play the dual role in our proposal. First, they play the same role of the CRs which are normally assumed to initiate the lightning processes. Secondly, they emit highly energetic particles being recorded as TASD bursts. This represents a key element why mysterious bursts [1, 2] which are associated with lightning events can be indeed originated from the AQNs which happen to appear in the area under the thunderclouds. The event rate estimated in [3] is based on this assumption.

Our presentation is organized as follows. In next Sect. 2 we overview the basic ideas of the AQN model, in Sect. 3 we overview the TA bursts observations [1, 2] and the basic ideas of the proposal [3] with the resolution of these puzzling bursts in terms of the AQN annihilation events. In Sects. 4 and 5 we argue that the emission of the radio pulse is inevitable consequence of the proposed mechanism, and we estimate its numerical characteristics. In Sects. 6 and 7 we estimate the radio pulse due to the Earth’s magnetic field. In Sect. 8 we explain how the conventional radio signals induced by the lightnings could be easily discriminated from the AQN-induced radio pulses. Finally, Sect. 9 is our conclusion where we suggest to search for a synchronized radio pulse with TA burst.

## The AQN DM model

The AQN model was invented two decades ago [4] to explain the observed similarity between the dark matter and the visible matter densities in the Universe.^1^ In this model, DM is made out of macroscopic lumps of quarks (or antiquarks) in colour superconducting (CS) phase with characteristic mass and size of order grams and $0.1\,\mathrm{\upmu m}$ respectively. In the AQN framework the baryogenesis is actually a charge segregation (rather than charge generation) process when the global baryon number of the universe remains zero at all times. Formation of AQNs relies on the coherent ${\mathscr {C}}{\mathscr {P}}$-violating axion field in early Universe, and consequently it leads to an asymmetry between matters and antimatter nuggets, which results in asymmetry between visible matter and antimatter in the observable Universe.

Unlike many conventional DM candidates such as weakly interacting massive particles (WIMPs) and axion, the AQN model naturally explains the observed similarity between the dark and visible densities in the Universe, i.e. $\varOmega _{\mathrm{DM}}\sim \varOmega _{\mathrm{vis}}$, with no fitting parameters, as both DMs and visible matters share the same QCD origin. As its name suggests, the AQN is similar to Witten’s quark nugget, see [5–7], and review [8], in many respects. This type of DM is in fact strongly-interacting by virtue of its macroscopic size, but it serves as the DM due to its diminutive number density.

In contrast to Witten’s original proposal, formation of AQNs does not rely on a first order QCD phase transition. Rather, accumulation and compression of baryon (or antibaryon) charge come from oscillations of axion domain wall (DW) bubbles during the QCD transition in early Universe which can squeeze the quark matter inside the bubbles. Due to the surface tension of the DW, ultimately the baryon (or antibaryon) charge trapped in a bubble is squeezed into a quark (or antiquark) nugget in CS phase. Because the CS phase is far more stable than the conventional hadronic phase, AQNs do not suffer from the evaporation problem, in contrast with the original Witten’s proposal [5–8]. We refer to the original papers [9–12] and a brief review [13] devoted to the specific questions related to the AQN’s formation, generation of the baryon asymmetry, and survival pattern in early Universe. We also refer to independent analysis [14] supporting the basic elements on the formation and survival pattern of the AQNs during the early stages of the evolution, including the BBN and CMB epochs.

For the present studies, however, we take the agnostic viewpoint, and assume that such nuggets made of antimatter are present in our Universe today irrespective to their formation mechanism. This assumption is consistent with all presently available cosmological, astrophysical and terrestrial constraints as long as the average baryon charge of the nuggets is sufficiently large as we review below.

The AQN DM model is consistent with all presently available cosmological, astrophysical and terrestrial constraints. The strongest direct detection limit is set by the IcuCube observatory, see Appendix A in [15]:

1 $$\begin{aligned} \langle B \rangle > 3\times 10^{24} \quad (\text {direct non-detection constraint}) . \end{aligned}$$

Similar limits are from the ANITA experiment and geothermal constrains which are also consistent with (1) as estimated in [16]. One soft constraint comes from the indirect non-detection of etching tracks in ancient mica [17], as it gives a more stringent limit in the available parameter space of DM nuggets with mass $M>55\,\mathrm{g}$, or correspondingly $B\gtrsim 10^{25}$ in the AQN model. However this constraint is based on an oversimplified assumption that all nuggets have equal mass, which is invalid in the AQN model since the AQN’s formation mechanism implies a reasonably broad distribution of nugget’s size. It is also claimed in [18] that the AQNs cannot account for more than 20$\%$ of the DM density according to the neutrino flux limits in the 20–50 MeV range observed from Super-Kamiokande (SuperK). However, as pointed out in [19], the claim [18] is based on an incorrect assumption that AQNs produce a neutrino spectrum similar to the conventional baryon-antibaryon annihilation events.^2^ Rather, the annihilation processes in CS phase are dramatically different from the conventional scenarios [19] because the energy scale of the lightest pseudo Goldstone mesons (pions and kaons) is in 20 MeV range, rather than 140 MeV in hadronic phase. The resulting neutrino spectrum features in CS phase computed in [19, 20] are consistent with the observations.

The presence of the *antimatter* nuggets in the system implies that there will be annihilation events leading to large number of observable effects on different scales: from galactic scales to the terrestrial rare events. In fact, there are many hints suggesting that such annihilation events may indeed took place in early Universe as well as they are happening now in present epoch. In particular, the AQNs might be responsible for a resolution of the “Primordial Lithium Puzzle” [21] during BBN epoch. The AQNs may also alleviate the tension between standard model cosmology and the recent EDGES observation of a stronger than anticipated 21 cm absorption feature as argued in [22]. The AQNs may also explain a recently observed “exotic” diffuse UV radiation in our galaxy [23] with very puzzling features, which are very hard to interpret within conventional astrophysical models [24]. The AQNs might be also responsible for famed long standing problem of the “Solar Corona Mystery” [25, 26] when the so-called “nanoflares” conjectured by Parker long ago [27] are identified with the annihilation events in the AQN framework. The AQNs could be also responsible for other mysterious and anomalous CR like events (along with [3] we already mentioned). It includes a mysterious anomalous events with noninverted polarity observed by the Antarctic Impulse Transient Antenna (ANITA) collaboration [28], and Multi Modal Clustering anomalous events [29] observed by the HORIZON 10T collaboration.

We conclude this short overview of the AQN model by emphasizing that the model is consistent with all present constraints as long as average baryon charge of the nuggets satisfies the relation (1). Furthermore, with the same set of parameters within the same framework it may explain a large number of mysterious phenomena listed above, which apparently suggest that the DM might be indeed in form of the mater and antimatter quark nuggets. In what follows we assume that such antimatter nuggets exist and together with nuggets saturate the DM density today.

## TA bursts as the AQN annihilation events under thunderclouds

The TA is designed for detection of extensive air showers induced by ultrahigh energy cosmic rays (CRs) consisting of 507 ground surface particle detectors (SDs) and 3 atmospheric fluorescence telescope stations. The TA collaboration have reported 10 excessively unlikely bursts of CR-like events are observed by the SDs [1, 2]. Comparing to conventional CRs, the TA burst events:

1. have a much smaller dispersion in reconstructed air shower fronts [see Fig. 3 and Fig. 4 in [1]), and do not have sharp edges in waveforms, the so-called *“curvature” puzzle*;
2. are temporally clustered within 1 ms that is highly unlikely (chance coincidence less than $$10^{-4}$$ for five-year observation) for ultrahigh energy CRs in the fitted energy range $$(10^{18}$$–$$10^{19}) $$ eV, the so-called *“clustering” puzzle*;
3. are all recorded under thunderstorm, and most of them are “synchronized” (less than 1 ms) or “related” (less than 200 ms) with the lightning events, the so-called *“synchronization” puzzle*,

see original articles [1, 2] and short reviews in [3, 13] for technical details.

Proposal [3] suggested the TA bursts can be a natural consequence of an *antimatter* AQN traversing through the thundercloud, where about $10^9$ weakly bound positrons are emitted from the AQN instantaneously in presence of the strong intracloud electric field ($\sim$ kV/cm) at altitude above 10 km.

The key mechanism in proposal [3] may be summarized as follows. First, the electric field ${\mathscr {E}}$ in the thunderclouds is characterized by the following parameters [30, 31]

2 $$\begin{aligned} {{\mathscr {E}}}\simeq \frac{\mathrm{kV}}{\mathrm{cm}}, \quad l_a \simeq 100 ~\mathrm{m},\quad \tau _{{\mathscr {E}}} \simeq \frac{l_a}{c}\simeq 0.3 \, \upmu \mathrm{s} \end{aligned}$$

where $l_a$ is the so-called avalanche length. The electric field is sufficiently strong to ionize an *antimatter* AQN and induce liberation of weakly bound positrons:

3 $$\begin{aligned} \varDelta E \simeq [e{{\mathscr {E}}}\cdot R_{\mathrm{cap}}] \sim 2 \hbox {keV}\gtrsim \hbox {E}_{\mathrm{bound}} , \end{aligned}$$

where $E_{\mathrm{bound}}\sim \mathrm{keV}$ is the binding energy of the positrons, and $R_{\mathrm{cap}}\sim 2\,\mathrm{cm}$ is the typical distance (from the nugget’s core where positrons reside [3].

These liberated positrons will be accelerated to MeV energies in the background of electric field characterized by typical length scale $l_a \simeq 100$ m according to (2):

4 $$\begin{aligned} E_{\mathrm{exit}}\simeq [e{{\mathscr {E}}} \cdot l_a]\sim 10 \, \hbox {MeV} . \end{aligned}$$

Thereafter, the positrons exit the region of strongly fluctuating electric field which is known to be present under thunderclouds.

**Fig. 1:**
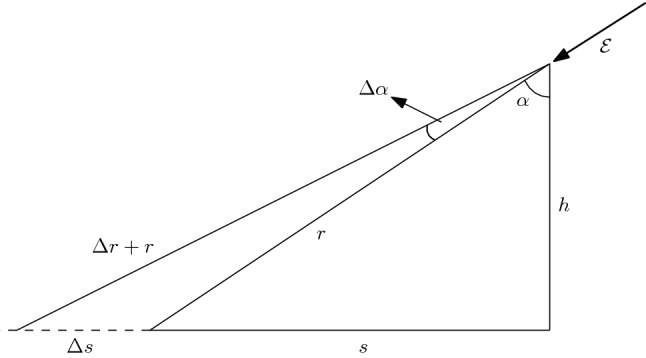
The positrons move along the cone with angle $\varDelta \alpha$ and inclination angle $\alpha$ with respect to the vertical direction. The angular spread $\varDelta \alpha \ll \alpha$ is assumed to be small. The spatial spread on the surface is determined by $\varDelta s$, while the additional travelling path is determined by $\varDelta r$, see estimates in the text. The altitude is assumed to be within conventional range $h\simeq$ (4–12) km. Instant direction of the electric field ${\mathscr {E}}$ at the moment of exit of the positrons is also shown. Figure adapted from [3]

Comparison to conventional CR air shower, the positron flux induced by AQN has a smaller dispersion angle $\varDelta \alpha$ and spatial spread $\varDelta s$: [3]

5 $$\begin{aligned}&\varDelta s \simeq r \left( \frac{\varDelta \alpha }{\cos \alpha }\right) \simeq \frac{1~\mathrm{km}}{\cos \alpha } \left( \frac{r}{10~\mathrm{km}}\right) \left( \frac{\varDelta \alpha }{0.1}\right) , \nonumber \\&\varDelta r \simeq \varDelta s \sin \alpha , \quad \varDelta \alpha \simeq \left( \frac{v_{\perp }}{c}\right) \in (0\text {--} 0.1) , \end{aligned}$$

where we choose the transverse component (with respect to the electric field) of the velocity $v_{\perp }\simeq \sqrt{2 \varDelta E/m}\simeq 0.1c$, see Fig. 1 for precise definitions of the parameters. This behaviour^3^ is consistent with feature **1** coined as the *“curvature” puzzle*.

A TA burst in the AQN framework represents the cluster of events related to one and the same AQN which traverses through the distance $$L_{\mathrm{burst}}\simeq 250\,\mathrm{m}$$ during $$\varDelta t\simeq 1\,\mathrm{ms}$$ at relativistic speed, rather than a “coincidental” combination of independent CR events. It also resolves the dramatic inconsistency (when interpreted in terms of the CR air showers) for the burst events when the event rate favours an energy range in $$10^{13} $$ eV, but the intensity of the events suggests $$(10^{18}$$–$$10^{19}) $$ eV, which explains the item **2** coined as the *“clustering” puzzle.*

Lastly, an AQN can serve as a trigger of runaway breakdown avalanche as discussed in [3].^4^ This explains the strong correlation between bursts and lightning events within AQN interpretation. The AQN-induced burst may or may not initiate lightnings depending on presence of other required ingredients for runaway breakdown avalanche to start.

To rephrase it, an AQN plays the dual role when it propagates in the thunderclouds: it emits the very energetic positrons, and it may also trigger the lightning, similar to conventional CR. The energetic positrons is the source for TASD bursts, while the feature of triggering explains the observed correlation between the bursts and lightnings in the AQN framework. It explains feature **3** coined as the *“synchronization” puzzle*, as well as it answers the question of why most but not all bursts are correlated with lightnings.

To summarize this section: the mysterious bursts (with highly unusual features **1–3** as listed at the beginning of the section) CR-like events observed by TASD [1, 2] are naturally interpreted as the cluster events generated by the AQNs propagating in thunderstorm environment. Some features of these events (such as intensity and the basic normalization factors) suffer inevitable uncertainties due to very complex dynamical properties of the system (the AQN propagation under thunderstorm with large Mach number). However, there are some features of the system such as given by Eq. (5) which are not sensitive to these uncertainties and represent almost model-independent consequences of the proposal [3].

## Radio pulse and TA burst as synchronized events

This section is devoted to studies of the radio signals which always accompany TA bursts when interpreted in terms of the AQN annihilation events under the thunderstorm as presented in previous Sect. 3. We shall argue below that the emission of the radio pulse is inevitable consequence of the proposed mechanism. Furthermore, the radio pulse must be synchronized with TA burst irrespective of whether the bursts are related or unrelated to the lightning events. This synchronization must be within $10 \, \upmu \mathrm{s}$ from every TA event as the positrons and radio waves originated from the same location at the same instant and both propagate with the speed of light. As the typical time and spatial spreads of the TA events do not exceed $10 \, \upmu \mathrm{s}$, the synchronization must be within the same temporal and spatial range.

This section is organized as follows. In next Sect. 4.1 we study the trajectory of the liberated positrons in a typical electric field characterized by parameters (2). We analyze the total intensity and the angular distribution of the radio emission in Sect/ 4.2, while the spectral properties of the pulse are studied in Sect. 4.3. Finally, in Sect. 4.4 we study the properties of the electric field’s pulse which can be detected at the surface detector area.

### Positron’s acceleration in electric field under thunderstorm

Our goal here is to describe the dynamics of the liberated positrons which are emitted with initial energies in keV range according to Eq. (3). To simplify things we assume that the electric field ${{\mathscr {E}}}$ directed along $\mathbf {z}$-axis is uniform and characterized by parameters (2). In this case the solution is well known [37] and can be described as follows. At the very initial moment of acceleration at $t\ll t_0$ the particle moves with non-relativistic velocities according to conventional formulae:

6 $$\begin{aligned}&z(t)\simeq \frac{p_z^0}{m}t+\frac{|e|{{\mathscr {E}}} }{2m}t^2, \quad t\ll t_0\equiv \frac{mc}{|e|{{\mathscr {E}}} } \nonumber \\&x_{\perp }(t)\simeq \frac{p_{\perp }^0}{m}t, \quad \frac{p_{\perp }^0}{m}\simeq \frac{p_z^0}{m} \simeq \frac{v_{\perp }}{c} , \end{aligned}$$

where $p_z^0\sim p_{\perp }^0$ is the initial positron’s momentum at the moment of liberation from AQN. Numerically, this non-relativistic portion of the positron’s journey is very short as $t_0\sim 10^{-8} \, \mathrm{s}$. It will be ignored in all our discussions which follow.

The portion of the positron’s journey which plays the key role in our discussions is the motion with constant acceleration $e{{\mathscr {E}}}/m$ and the velocity close to *c*. The corresponding motion is determined by the formulae:

7 $$\begin{aligned}&z(t)= \frac{c}{|e|{{\mathscr {E}}} }\left[ \sqrt{(p_{\perp }^0)^2 +m^2c^2+(p_z^0+|e|{{\mathscr {E}}} t)^2}-\frac{E^0}{c}\right] , \nonumber \\&x_{\perp }(t)\simeq \frac{cp_{\perp }^0}{|e|{{\mathscr {E}}} } \ln \frac{2|e|{{\mathscr {E}}} t}{mc}, \quad t\gg t_0\equiv \frac{mc}{|e|{{\mathscr {E}}} } \end{aligned}$$

where $E^0\simeq (mc^2+\varDelta E)$ is the total initial energy of positrons at the moment of liberation.

The most important observation here is that the positrons move with ultra-relativistic velocities for most of the time, and the displacement in transverse direction (with respect to the orientation of the electric field) is very modest such that the majority of the particles remain in the system and continue their acceleration for the entire time interval $\tau _{{\mathscr {E}}}\sim 0.3 \,\upmu \mathrm{s}$ determined by parameter $l_a$ according to (2). At this point most of the particles assume very high energy close to 10 MeV according to Eq. (4) while transverse momentum distribution is characterized by $(v_{\perp }/c)\lesssim 0.1$. The elastic scattering of the positrons off the atmospheric molecules during this short journey plays very minor role as the kinetic energy of positrons being in keV range at the initial instant assumes MeV energies very quickly such that the corresponding cross section never becomes sufficiently large to modify the positron’s dynamics as computed above in vacuum.

### Intensity and the angular distribution

Our next task is to present conventional formulae for the intensity and the angular distribution of the radio emission due to the relativistic positrons (7) accelerating in electric field ${\mathscr {E}}$ with parameters (2). As we discussed above the velocity $\mathbf {v}$ of the positrons is mostly oriented along the electric field while $v_{\perp }$ is small. Therefore, we assume that ${\mathscr {E}} \parallel \mathbf {v}$ to simplify our formulae which follow. In this simplified geometry the angular distribution for the intensity assumes the following form, see e.g. [37, 38]:

8 $$\begin{aligned} \frac{dI (t)}{d\varOmega }\simeq \frac{N^2e^2 \mathbf {a}^2 (t)}{4\pi c^3}\frac{\sin ^2\theta }{(1-\frac{v}{c}\cos \theta )^5}, ~~~ \mathbf {a}\equiv \frac{e {\mathscr {E}}}{m\gamma ^3}, \end{aligned}$$

where *N* is the number of coherent positrons participating in the radio wave emission, to be estimated below. The angle $\theta$ in this expression is defined as usual as the angle between the velocity $\mathbf {v}$ of the charged particle at the moment of emission and the direction $\mathbf {n}$ of the observer, i.e. $\mathbf {v}\cdot \mathbf {n}=v\cos \theta$. Formula (8) explicitly shows that the emission mostly occurs along ${\mathscr {E}}$ direction as ${\mathscr {E}} \parallel \mathbf {v}$. This is precisely the same direction where burst events are recorded as shown on Fig. 1.

Integration over all angles leads to well known expression for the total intensity:

9 $$\begin{aligned} I(t)\simeq \frac{2N^2e^2 }{3c^3} \left[ \mathbf {a}^2(t)\gamma ^6\right] , ~~~ \gamma \equiv \frac{1}{\sqrt{(1- {v^2}/{c^2})}} \end{aligned}$$

Formula (9) represents the well known feature of the emission that the intensity is strongly enhanced for ultra-relativistic particles, which is obviously the case for positrons accelerated up to 10 MeV energies according to Eq. (4). This implies that the relativistic enhancement factor entering Eq. (9) is enormous: $(E_{\mathrm{exit}}/ m)^6\sim 10^8$.

### Spectral characteristics of the radio emission

The main goal of this subsection is to understand the spectral characteristics of the radio emission. It will also allow to estimate the coherence factor *N* entering Eqs. (8) and (9) as this factor obviously depends on the frequency of radiation and corresponding wave length.

The starting point for these studies is spectral property of the electric field $\mathbf {E}_{\omega }$ emitted by the accelerating positrons, see e.g. [37, 38]:

10 $$\begin{aligned} \mathbf {E}_{\omega }=\int _{-\infty }^{+\infty } \mathbf {E}e^{i\omega t}dt, ~~ \mathbf {E} =\frac{Ne}{c^2R}\frac{\mathbf {n} \times \left( (\mathbf {n}-\frac{\mathbf {v}}{c}\right) \times \mathbf {a})}{(1-\frac{\mathbf {n}\cdot \mathbf {v}}{c})^3}, \end{aligned}$$

where all quantities at the right hand side of Eq. (10) must be computed as the retarded times $t'$:

11 $$\begin{aligned} t'\approx t-\frac{R_0}{c}+\frac{\mathbf {n}\cdot \mathbf {v}t'}{c} \rightarrow t=t' \left( 1-\frac{\mathbf {n} \cdot \mathbf {v}}{c}\right) +\frac{R_0}{c}, \end{aligned}$$

where we assumed that the positrons move with approximately constant time-independent velocity $|\mathbf {v}|\approx c$. This assumption allows us to represent the Fourier component $\mathbf {E}_{\omega }$ in the following form

12 $$\begin{aligned} \mathbf {E}_{\omega }= \frac{e^{ikR_0}}{R_0} \left( \frac{Ne}{c^2}\right) \left( \frac{\omega }{\omega '}\right) ^2 \left[ \mathbf {n}\times \left( (\mathbf {n}-\frac{\mathbf {v}}{c}) \times \mathbf {a}_{\omega '}\right) \right] , \end{aligned}$$

where $\omega '$ and $\mathbf {a}_{\omega '}$ are defined as follows

13 $$\begin{aligned} \omega '\equiv \omega \left( 1-\frac{\mathbf {n} \cdot \mathbf {v}}{c}\right) , ~~~ \mathbf {a}_{\omega '} =\int _{-\infty }^{+\infty }\mathbf {a(t')}e^{i\omega ' t'}dt' \end{aligned}$$

which precisely the combination entering the Fourier transform (10).

Using Fourier component for $\mathbf {E}_{\omega }$ as given by Eq. (12) and magnetic component $\mathbf {B}_{\omega } =i(\mathbf {k}_{\omega }\times \mathbf {E}_{\omega })$ one can compute the energy $dE_{\mathbf {n}\omega }$ emitted into solid angle $d\varOmega$ with frequency interval $d\omega$ [37, 38]:

14 $$\begin{aligned} \frac{dE_{\mathbf {n}\omega }}{d\varOmega d\omega } =\left( \frac{N^2 e^2}{4\pi ^2 c^3}\right) \left( \frac{\omega }{\omega '}\right) ^4 \left| \mathbf {n}\times \left( (\mathbf {n} -\frac{\mathbf {v}}{c})\times \mathbf {a}_{\omega '}\right) \right| ^2. \end{aligned}$$

One can simplify this expression by assuming that the $\mathbf {a}\parallel \mathbf {v}$ as we previously discussed. Furthermore, one can carry out the integration over $d \varOmega$ by integrating over $d \omega '$ for a given $\omega$ as these two variables are related according to (13). Indeed, the emission is mostly concentrated along the cone with $\theta ^2\approx (1-v^2/c^2)$. Furthermore,

15 $$\begin{aligned} \omega '\equiv \omega \left( 1-\frac{\mathbf {n} \cdot \mathbf {v}}{c}\right) \approx \frac{\omega }{2} \left[ \frac{1}{\gamma ^2} + {\theta ^2} \right] , \end{aligned}$$

where we expanded $\cos \theta \approx (1-\theta ^2/2)$ and represented small factor $(1-v/c)\approx {1}/{(2\gamma ^2)}$ in terms of the conventional combination $\gamma$ which is valid approximation for ultra-relativistic positrons with $|\mathbf {v}|\simeq c$. Now, integration over angles $d \varOmega$ can be replaced by integration over $d\omega '$ as these two variables are related by (15). Therefore,

16 $$\begin{aligned} d\varOmega =2\pi \sin \theta d \theta \approx 2\pi \left( \frac{d\theta ^2}{2} \right) \approx 2 \pi \left( \frac{d\omega '}{\omega } \right) \end{aligned}$$

such that the energy $dE_{\omega }$ emitted within the frequency interval $d\omega$ assumes the form

17 $$\begin{aligned} \frac{dE_{\omega }}{ d\omega } = \left( \frac{N^2 e^2\omega ^2}{2\pi c^3}\right) \int _{\frac{\omega }{2\gamma ^2}}^{\infty } \frac{d\omega ' |\mathbf {a}_{\omega '}|^2}{(\omega ')^3} \left[ 2-\frac{\omega }{\omega ' \gamma ^2} \right] , \end{aligned}$$

where the low limit of integration is determined by $\theta =0$ in relation (15).

The same expression can be thought as the angular distribution of the emission

18 $$\begin{aligned} \frac{dE_{\omega }}{ d\omega }= \left( \frac{N^2 e^2\gamma ^6 }{2\pi c^3}\right) \frac{16|\mathbf {a}_{\omega '}|^2}{(1+\gamma ^2\theta ^2)^3}\left[ \frac{\gamma ^2\theta ^2}{1+\gamma ^2\theta ^2} \right] \cdot \frac{d\varOmega }{2\pi }, \end{aligned}$$

where $|\mathbf {a}_{\omega '}|$ should be expressed in terms of $\theta$ according to relation (15).

Our next task is to model the acceleration $\mathbf {a}(t)$ for a typical thunderstorm electric field with parameters (2). The corresponding Fourier transform (13) determines $\mathbf {a}_{\omega '}$ which enters expression for ${dE_{\omega }}/{ d\omega }$ as given by Eq. (17). Our simplest possible choice is as follows:

19 $$\begin{aligned} \mathbf {a}(t)\approx \frac{e {\mathscr {E}}}{\gamma ^3 m}, ~~~ t\in (0, \tau _{{{\mathscr {E}}}} ), \end{aligned}$$

while $\mathbf {a}(t)\approx 0$ for *t* being outside of this interval. The corresponding expression for $\mathbf {a}_{\omega '}$ defined by (13) assumes the form

20 $$\begin{aligned} | \mathbf {a}_{\omega '}|^2=\frac{4e^2{{\mathscr {E}}}^2}{m^2\gamma ^6\omega '^2}\sin ^2\left( \frac{\omega ' \tau _{{{\mathscr {E}}}}}{2}\right) . \end{aligned}$$

Now we can perform the integration $d\omega '$ in Eq. (17). For our simple model for acceleration (19) the integral can be approximately computed for small $(\omega ' \tau _{{{\mathscr {E}}}})\ll 1$. This leads us to the order of magnitude estimate for ${dE_{\omega }}/{ d\omega }$:

21 $$\begin{aligned} \frac{dE_{\omega }}{ d\omega }\approx \left( \frac{N^2 e^2 }{2\pi c^3}\right) \cdot \left( \frac{e^2{{\mathscr {E}}}^2 \tau ^2_{{{\mathscr {E}}}}}{m^2\gamma ^6}\right) \cdot \left( \frac{4\gamma ^4}{3}\right) . \end{aligned}$$

The same expression can be derived from Eq. (18) by integrating over the angles $d\varOmega$.

This spectral density holds as long as $\omega$ is sufficiently small. To be more precise:

22 $$\begin{aligned} \omega \lesssim { \gamma ^2}{\tau ^{-1}_{{{\mathscr {E}}}}}, \end{aligned}$$

which represents the dominant contribution to the integral (17) for small $\omega$. The emission with higher frequencies will be power-suppressed as $\omega ^{-2}$.

Few comments are in order. The spectral density (21) integrated over all angles $d\varOmega$ approximately constant and does not depend on $\omega$ as long as condition (22) is satisfied. This is of course a well-known feature of the constant acceleration (19) which we used as a simplified model for the electric field. In reality, the electric field under thunderstorm obviously fluctuates in time and space. This will obviously modify the spectral features (21). However, we expect that a typical frequency of the emission as given by (22) will hold because it is basically determined by the typical time scale of the problem $\tau _{{{\mathscr {E}}}}$ and expected relativistic factor $\gamma$ which is not very sensitive to the details of the fluctuating electric field ${{\mathscr {E}}}$.

The crucial observation here is that the typical frequency of emission is not simply given by the scale $\tau ^{-1}_{{{\mathscr {E}}}}$ as one could naively expect based on dimensional arguments. Rather, the emission extends to much broader region due to the large relativistic factor $\gamma ^2$ as inequality (22) states.

We postpone for the detail numerical estimates to Sect. 5. Now we want to make few simple numerical estimates supporting the main claim of this work that the frequency of emission is in the radio band $\nu \in$ (0.5–200) MHz. Furthermore, the emission is mostly oriented along the same direction where TA bursts are recorded. Therefore, one should anticipate a strong synchronization between the TA bursts and the radio pulses as both emission occurs at the same location at the same instant, and propagate to the corresponding detectors with the same speed of light. These features are not very sensitive to details of the dynamics of the AQNs, nor specific features of electric field under thunderstorm. Rather, all these features are inevitable consequences of the basic AQN framework along with pure geometrical properties of these observables.

With these comments in mind we can represent the typical band as follows

23 $$\begin{aligned} \nu \lesssim \frac{{ \gamma ^2}}{2\pi {\tau _{{{\mathscr {E}}}}}} \approx 200~ \mathrm{MHz},\quad \nu \equiv \frac{\omega }{2\pi }, ~~~ \gamma \approx 20, \end{aligned}$$

which we expect to hold irrespective of specific features of the AQN model as it is entirely determined by well established typical time scales of the electric field under the thunderstorm (2). One could naively think that the typical frequency of emission could be extrapolated to very low $\nu$ as the spectral density (21) apparently does not depend on frequency. This is not quite correct conclusion though as we shall discuss in next Sect. 5.

### Radio pulse of the electric field

The goal of this section is to estimate the intensity of the electric field (10) at very large distances *R* where it can be potentially detected. The orientation of $\mathbf {E}$ field is determined by cross product (10) where one can assume (for the simplicity of the numerical estimates) that $\mathbf {v}\parallel \mathbf {a}$, similar to our previous estimates for the spectral intensity of the emission (17). The absolute value for $|\mathbf {E}|$ at large distances can be estimated as follows

24 $$\begin{aligned} |\mathbf {E}|\approx \frac{Ne |\mathbf {a}| \theta }{c^2R} \left( \frac{\omega }{\omega '}\right) ^3 \approx \frac{Ne |\mathbf {a}| \theta }{c^2R} \left( \frac{2\gamma ^2}{1+\gamma ^2\theta ^2}\right) ^3, \end{aligned}$$

where *R* is the distance from the emission site to the area where the electric field $|\mathbf {E}|$ could be recorded. It should not be confused with parameter *r* which enters all formulae from Sect. 3 and describes the distance from the emission site to the SD site where the energetic positrons could be detected. Numerically these parameters are similar, of course. However, the radio signal can be observed in a different location from the local area where the positrons hit the TASD.

This formula explicitly shows that the time duration of the radio pulse and its properties are unambiguously determined by the features of the electric field ${\mathscr {E}}$ under thunderstorm. For simple model (19) this implies that the radio pulse lasts $\tau _{{\mathscr {E}}}$ while the absolute value of the pulse is estimated as

25 $$\begin{aligned} |\mathbf {E} (\theta ,t)| \approx \frac{Ne \theta }{c^2R} \left( \frac{2\gamma ^2}{1+\gamma ^2\theta ^2}\right) ^3 \left( \frac{e{\mathscr {E}}(t)}{m \gamma ^3}\right) , \quad t\in (0, \tau _{{{\mathscr {E}}}} ) . \end{aligned}$$

It is important to emphasize that the duration of the pulse $\tau _{{{\mathscr {E}}}}$ is not very sensitive to the details of the AQN model as it is entirely determined by well established typical time scales of the electric field under the thunderstorm (2). This feature is very similar to our previous arguments regarding a typical frequency of the radio emission (23). At the same time the intensity of the pulse is highly sensitive to the details of the AQN model as it depends on the number of positrons *N* participating in the emission. In this respect this feature of insensitivity to any specific details of the AQN model for the pulse duration $\tau _{{{\mathscr {E}}}}$ and the frequency $\nu$ as given by (23) is similar to our studies of the TA burst events when such features as the “curvature”, the timing and the spatial spread of the bursts are not sensitive to the details of the AQN model, but entire determined by the geometry as reviewed in Sect. 3. This should be contrasted with estimations [3] of the intensity of the TA bursts which are highly sensitive to specific features of the model, which are hard to carry out in a quantitative manner.

## Numerical estimates

Our goal here is to present some numerical results using parameters which had been used in our previous estimations [3] related to puzzling TASD bursts as reviewed in Sect. 3. One should emphasize from the very beginning that the corresponding estimates suffer from huge uncertainties, and should be considered as the order of magnitude estimates, at the very best. Our main arguments of the present work are not based on these highly model dependent estimates. Rather, our main arguments are based on essentially model-independent features of the radio emission such as typical frequency band (23) and strong synchronization between TASD burst events and the radio emission. Nevertheless, we think that an order of magnitude estimates for the amplitude of the electric field as presented below could be useful as they demonstrate the consistency of the proposal. The same estimates also demonstrate the principle feasibility to detect such radio signals.

**Fig. 2:**
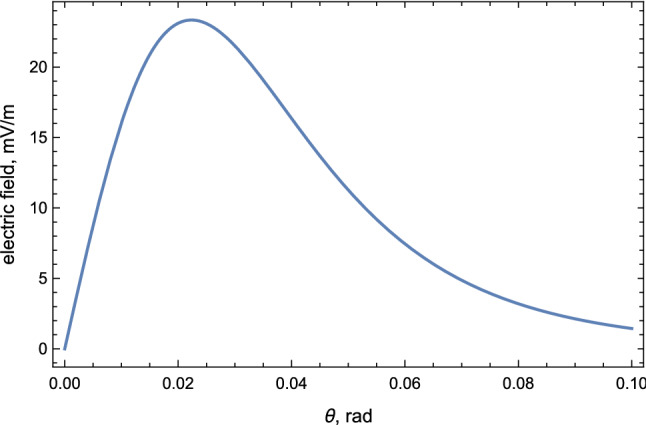
Strength of electric field (26) induced under thunderstorm versus observation angle $\theta$. The parameters are chosen to be $N=10^9$, $\gamma =20$, ${{\mathscr {E}}}=1\,\hbox {kV}/\hbox {cm}$

We start with numerical estimation of the strength of the electric field of the pulse (25) in conventional units $(\hbox {mV}/\hbox {m})$:

26 $$\begin{aligned}&|\mathbf {E} (\theta ,t)| \approx 90 \frac{\mathrm{mV}}{\mathrm{m}} \cdot \left[ \frac{(\gamma \theta )}{(1+\gamma ^2\theta ^2)^3}\right] \nonumber \\&\quad \cdot \left( \frac{\gamma }{20 } \right) ^2\cdot \left( \frac{N}{10^9}\right) \cdot \left( \frac{10~\mathrm{km}}{R}\right) , \quad t\in (0, \tau _{{{\mathscr {E}}}} ) \end{aligned}$$

where factor *N* is the number of the coherent positrons participating in the emission, to be estimated below. The orientation of the electric field is determined by the cross product as given by (10), and it is approximately (up to small angle $\theta$) points along $\mathbf {a}$ which represents the direction of the electric field under thundercloud at the instant of emission. The temporal shape of the pulse (bipolar, unimodal or even more complicated form) is also determined by the same fluctuating electric field ${\mathscr {E}}$ at the moment of emission. The intensity of the field is strongly peaks along $\mathbf {n}$ with typical angle $\theta \gamma \lesssim 1$, see Fig. 2 for a precise angular distribution of $|\mathbf {E} (\theta )|$.

Our next task is the estimation of parameter *N* entering Eq. (26). Our estimates will be entirely based on the observations, rather than on theoretical computations which inevitably suffer from huge uncertainties as reviewed in Sect. 3. Therefore, the logic of our estimation of parameter *N* is as follows. Let us assume that the TASD bursts which have been recorded are indeed due to the positrons emitted by AQNs as proposed in [3]. In this case the number of detected particles must be proportional to the density of particle detectors. This number is known for presently operating 507 SD detectors with area $3\,\mathrm{m}^2$ each. These detectors are covering $680 ~\mathrm{km}^2$ total area. The number of detected particles must be also proportional to the area $\sim \pi (r\varDelta \alpha )^2$ where particles had been recorded by TASD during a single event within the burst. Therefore, the number of positrons *N* at the emission site can be estimated from the following relation:

27 $$\begin{aligned} N_{\mathrm{positrons}}^{\mathrm{detected}}[\varDelta s] \!\approx \! N \left[ \frac{ 507\cdot 3~\mathrm{m}^2}{680~\mathrm{km}^2} \right] \left[ \left\langle \exp \left( -\frac{r}{\lambda }\right) \right\rangle \right] \left[ \pi (r\varDelta \alpha )^2\right] \end{aligned}$$

In our estimate (27) the number of detected particles being recorded over the surface area $\pi (\varDelta s)^2$ [left hand side of (27)] varies for different events within a single burst, and it normally varies in the range (2–6) $\cdot 10^2$. In our estimate (27) we inserted the suppression factor $\langle \exp (-r/\lambda )\rangle \sim 0.1$ introduced in [3] to account for some attenuation of the positrons travelling a distance $r\sim$ 10 km or so when the mean free path $\lambda$ for positrons with few MeV energy is order of kilometre at the sea level and several kilometres at higher altitudes. One should also note that $\pi (r\varDelta \alpha )^2$ on the right hand side of Eq. (27) is not identically the surface area $\sim \pi (\varDelta s)^2$ where particles are being recorded. These areas are directly related to each other through angle $\alpha$ according to Eq. (5), see Fig. 1 for notations.

The relation (27) suggests that number *N* entering Eq. (26) for $(r \varDelta \alpha ) \sim 1 ~\mathrm{km}$ (which corresponds to a typically observed spatial spread for the burst events) can be estimated as

28 $$\begin{aligned} N\approx (0.3\text {--}1)\cdot 10^9 \quad \mathrm{for} \, \, (r \varDelta \alpha ) \sim 1 ~\mathrm{km}. \end{aligned}$$

This estimate for *N* corresponds to the amplitude of the electric field (26) on the level $20\,(\hbox {mV}/\hbox {m})$ measured at distance $R\simeq 10$ km from the source of the emission within the angle range $(\theta \gamma )\lesssim 1$ where the most of the radio emission occurs.

**Fig. 3:**
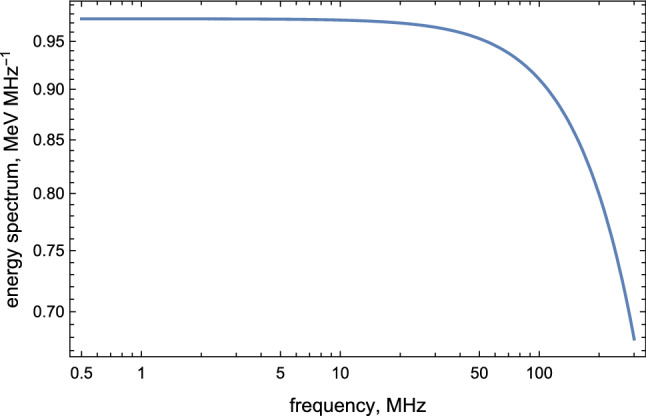
Spectrum (17) in terms of frequency $\nu$, thunderstorm radiation. The parameters are chosen to be $N=10^9$, $\gamma =20$, ${{\mathscr {E}}}=1\,\hbox {kV}/\hbox {cm}$

Our last task in this section is establishing the lower frequency bound for the radio emission. The spectral density (21) which is approximately a constant, naively suggests that the spectrum extends to arbitrary low frequencies. In fact, it is a premature conclusion as formula (21) was derived assuming a constant acceleration. In reality, a typical acceleration time is determined by (2) such that one should expect that $\nu \gtrsim (2\pi {\tau _{{{\mathscr {E}}}}})^{-1}\approx 0.5~\mathrm{MHz}$. Therefore, the AQN-induced $\sim 0.3\upmu$s pulse represents a radio emission in the bandwidth $\nu \in (0.5\text {--}200) ~\hbox {MHz}$ with the amplitude of the corresponding electric field of the order $|\mathbf {E} (\theta ,t)| \sim 20\,(\hbox {mV}/\hbox {m})$ at distance $R\sim 10 ~\hbox {km}$ as estimated above. Important feature of this spectrum is that it is very broad. Indeed, the intensity of the radiation changes by factor two or so when the frequency varies by two orders of magnitude. Another comment which follows from Fig. 3 is that the portion of the positron’s energy being converted into the radio waves is very tiny as initial positrons energy can be estimated as $10 N~ \mathrm{MeV}$ when *N* is estimated in Eq. (28). This implies that the initial energy is many orders of magnitude greater than the radio wave energy shown on Fig. 3.

The crucial point of our analysis can be formulated as follows. This radio pulse must be synchronized with an event within TA burst within $10\,\upmu$s because the TA burst and the radio emission are originated from the same location emitted at the same instant and both signals propagate with the speed of light along the same direction to be detected within $\sim \hbox {km}^2$ area.

It is important to emphasize that such strong synchronization must hold even if the TA bursts are not related to any lightning events and can be easily discriminated from much stronger radio emission which always accompany lightnings. Therefore, such synchronization between the radio pulses and the TA bursts, if observed, can be easily distinguished from any other possible correlation between radio emission and conventional lightning events, see Sect. 8 for the details.

## Geosynchrotron radiation

Another significant radio emission is the geosynchrotron radiation which is due to presence of the Earth’s magnetic field ${{\mathscr {B}}}\sim 0.5\,\mathrm{gauss}$. This effect becomes important near the end of radio emission under thunderstorm. At this instant all the positrons are ultrarelativistic ($\gamma \sim 20$) but they are no longer accelerating as they already left the region of the external electric field ${\mathscr {E}}$. Trajectory of positrons are helical in presence of the magnetic field of Earth ${{\mathscr {B}}}\sim 0.5\,\mathrm{gauss}$. The acceleration in this case is perpendicular to the direction of velocity $\mathbf {v}\perp \mathbf {a}_{{\mathscr {B}}}$, so it is convenient to set up the following coordinate with respect to the direction of observer $\mathbf {n}$:

29 $$\begin{aligned} \hat{\mathbf {v}}\cdot \mathbf {n} =\cos \theta ,\quad \hat{\mathbf {a}}_{{\mathscr {B}}}\cdot \mathbf {n}=\sin \theta \cos \phi \end{aligned}$$

Radius of the helical trajectory can be estimated as

30 $$\begin{aligned}&\rho \approx \frac{c^2}{|\mathbf {a}_{{\mathscr {B}}}|} \approx \frac{0.7\,\mathrm{km}}{\sin \theta _{{\mathscr {B}}}} \left( \frac{\gamma }{20}\right) \left( \frac{0.5\,\mathrm{gauss}}{{{\mathscr {B}}}}\right) ,\nonumber \\&|\mathbf {a}_{{\mathscr {B}}}|\approx \frac{e{{\mathscr {B}}}c \sin \theta _{{\mathscr {B}}}}{\gamma m} , \end{aligned}$$

where $\theta _{{\mathscr {B}}}$ is defined to be the angle between direction of ${{\mathscr {B}}}$ and the velocity of positrons $\mathbf {v}$.

Radiation from an ultrarelativistic particle has a narrow emission angle $\theta \lesssim \gamma ^{-1}$, similar to our previous discussions. However, the pulse of radiation for geosynchrotron radiation is much shorter [37, 38]:

31 $$\begin{aligned} \tau _{{\mathscr {B}}} \approx \frac{\rho }{2\gamma ^3c} =\frac{0.14\,\mathrm{ns} }{\sin \theta _{{\mathscr {B}}}} \left( \frac{20 }{\gamma }\right) ^2 \left( \frac{0.5\,\mathrm{gauss}}{{{\mathscr {B}}}}\right) \end{aligned}$$

This interval is much shorter than the time scale $\tau _{{\mathscr {E}}}\sim 0.3\,\upmu \mathrm{s}$ discussed previously due to extra suppression factor $\sim \gamma ^{-3}$. The energy spectrum integrated over angle $d\varOmega$ is well known [37–39]:

32 $$\begin{aligned}&\frac{d E_\omega }{d\omega } =\frac{c\tau _{{\mathscr {B}}}}{2\pi \rho }\frac{\sqrt{3}N^2e^2}{c}\gamma \frac{\omega }{\omega _c}\int _{\omega /\omega _c}^{\infty }K_{5/3}(x)dx , \nonumber \\&\omega _c =\frac{3}{2}\gamma ^3\left( \frac{c}{\rho }\right) \simeq \frac{3}{4\tau _{{\mathscr {B}}}} , \end{aligned}$$

where $K_{5/3}(x)$ is the modified Bessel function, and $\omega _c$ is the critical angular frequency beyond which radiation becomes negligible. 32

The electric field of the geosynchrotron radiation follows from Eq. (10):

33 $$\begin{aligned} |\mathbf {E}|&=\frac{Ne|\mathbf {a}_{{\mathscr {B}}}|}{c^2R} \left( \frac{\omega }{\omega '}\right) ^2\sqrt{1-\frac{(\mathbf {n} \cdot \hat{\mathbf {a}}_{{\mathscr {B}}})^2}{\gamma ^2} \left( \frac{\omega }{\omega '}\right) ^2} \nonumber \\&\approx \frac{Ne^2{{\mathscr {B}}}\sin \theta _{{\mathscr {B}}}}{\gamma mcR} \left( \frac{2\gamma ^2}{1+\gamma ^2\theta ^2}\right) ^2 \sqrt{1-\left( \frac{2\gamma \theta \cos \phi }{1+\gamma ^2\theta ^2}\right) ^2} , \end{aligned}$$

The estimation of electric field strength holds as long as the positron current remains coherent. The coherent length $l_{\mathrm{coh}}$ can be estimated in what follows. The dispersion in velocity is $\delta v\sim v_\perp \lesssim 0.1c$ from Eq. (5). The condition of coherence requires the dispersion length $\delta l$ to be much less than the radius $\rho$ of trajectory:

34 $$\begin{aligned} \delta l \approx \frac{\delta v}{c}l_{\mathrm{coh}} \sim \frac{v_\perp }{c}l_{\mathrm{coh}} \ll \rho . \end{aligned}$$

It implies $l_{\mathrm{coh}}\sim \rho$ that is insensitive to details of local parameters such as $\gamma$ and ${{\mathscr {B}}}$, and the geosynchrotron radiation is only significant within the first cycle of rotation.

## Numerical estimates on geosynchrontron radiation

**Fig. 4:**
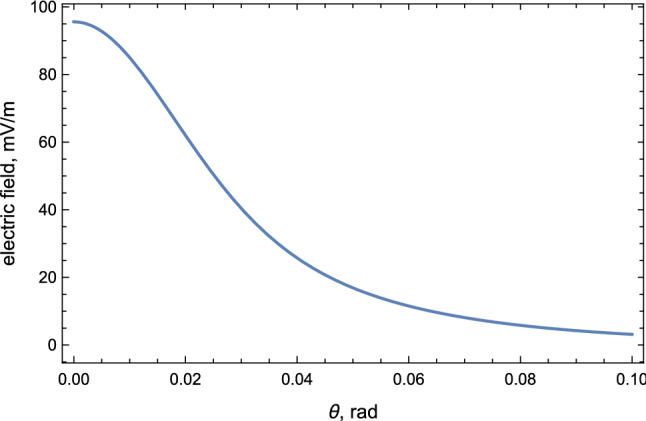
Strength of electric field (33) induced by geosynchrotron radiation versus obervation angle $\theta$. The parameters are chosen to be $N=10^9$, $\gamma =20$, ${{\mathscr {B}}}=0.5\,\mathrm{gauss}$, $\theta _{{\mathscr {B}}}=\phi =\pi /4$

Similar to Sect. 5, we make numerical estimation for the electric field strength and the frequency bandwidth. Numerical estimation for the strength of the electric field of pulse Eq. (33) in conventional units (mV/m) is given as:

35 $$\begin{aligned} |\mathbf {E}|&\approx 100 \,\frac{\mathrm{mV}}{\mathrm{m}} \cdot \frac{\sin \theta _{{\mathscr {B}}}}{(1+\gamma ^2\theta ^2)^2} \sqrt{1-\left( \frac{2\gamma \theta \cos \phi }{1+\gamma ^2\theta ^2}\right) ^2} \nonumber \\&\quad \times \left( \frac{\gamma }{10}\right) ^3 \left( \frac{N}{10^9}\right) \left( \frac{10\,\mathrm{km}}{R}\right) \left( \frac{{{\mathscr {B}}}}{0.5\,\mathrm{gauss}}\right) , \end{aligned}$$

see Fig. 4 for a precise angular distribution with specific parameters.

The upper limit of frequency band is determined by the critical frequency $\omega _c$:

36 $$\begin{aligned} \nu \lesssim \frac{\omega _c}{2\pi } =\frac{3}{8\pi \tau _{{\mathscr {B}}}} \approx 600 \,\mathrm{MHz} ,\quad \left( \theta _{{\mathscr {B}}}=\frac{\pi }{4}\right) , \end{aligned}$$

and the lower frequency cutoff is given by the fundamental frequency:

37 $$\begin{aligned} \nu \gtrsim \frac{c}{2\pi \rho }\approx {50\,\mathrm{kHz}} , \quad \left( \theta _{{\mathscr {B}}}=\frac{\pi }{4}\right) . \end{aligned}$$

**Fig. 5:**
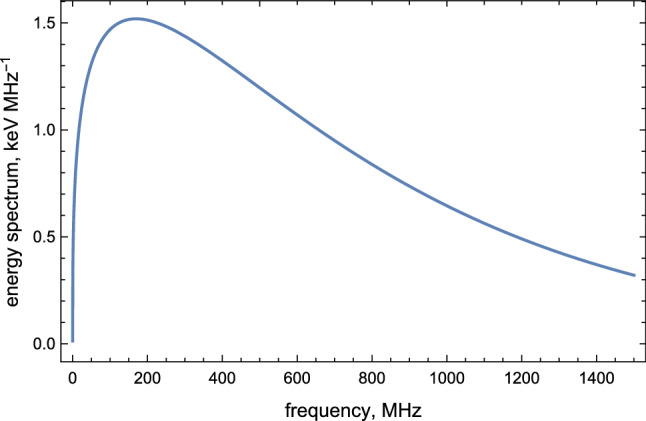
Spectrum (32) in terms of frequency $\nu$, geosynchrotron radiation. The parameters are chosen to be $N=10^9$, $\gamma =20$, ${{\mathscr {B}}}=0.5\,\mathrm{gauss}$, $\theta _{{\mathscr {B}}}=\pi /4$

Therefore, the geosynchrotron induces a radio pulse with time duration $\sim 0.14\,\mathrm{ns}$ in the bandwidth $\nu \in (0.05\text {--}600)\,\mathrm{MHz}$ (see Fig. 5) and the amplitude of the corresponding electric field is of order $\sim 50\,\hbox {mV}/\hbox {m}$ at distance $R\sim 10\,\mathrm{km}$.

While numerical values of the frequency bandwidth $\nu \sim (0.1\text {--}10^2)~ \hbox {MHz}$ and the maximal strength of electric field of the radio wave [$|\mathbf {E}|\sim (20 \text {--}50)~\hbox {mV}/\hbox {m}$] are similar in both cases of radio pulses (which we term as ${\mathscr {E}}$-type and ${\mathscr {B}}$-type correspondingly) there is an important distinct feature, which is manifested in dramatically different time durations in these two cases. To be more precise, a ${\mathscr {B}}$-type pulse induced by geosynchrotron radiation ($\tau _{{\mathscr {B}}}\sim 0.14\,\mathrm{ns}$) is much shorter than ${\mathscr {E}}$-type pulse with ($\tau _{{\mathscr {E}}} \sim 0.3\,\upmu \mathrm{s}$).

This distinct feature is in fact an absolutely crucial element for possible potential observations of the emission. This difference between $\tau _{{\mathscr {B}}}$ and $\tau _{{\mathscr {E}}}$ is the main reason for dramatically different energy injection which is measured in units $\hbox {MeV}\cdot \hbox {MHz}^{-1}$ in case of ${\mathscr {E}}$-type emission to be contrasted with much lower $\hbox {keV}\cdot \hbox {MHz}^{-1}$ scale in case of ${\mathscr {B}}$-type emission, see Figs. 3 and 5 correspondingly.

Therefore, the total energy of the radio emission integrated over the pulse time is much greater for the ${\mathscr {E}}$-type radiation in comparison with ${\mathscr {B}}$-type radiation. Furthermore, the required resolution to detect the very short ${\mathscr {B}}$-type pulse with $\tau _{{\mathscr {B}}} \sim \hbox {ns}$ requires much more demanding instruments than recording of the conventional ${\mathscr {E}}$-type pulses with duration time of order $\tau _{{\mathscr {E}}}\sim \upmu \mathrm{s}$. These arguments strongly suggest that ${\mathscr {E}}$-type radiation plays the dominant role in emission processes. Therefore, any future studies (including possible observations) should be focused on ${\mathscr {E}}$-type radiation analyzed in Sect. 5.

## Can conventional physics be responsible for proposed effects?

The question we address in this section can be formulated as follows. It has been known for quite sometime that the thunderstorm lightnings are always accompanied by the radio emissions, see e.g. reviews [30, 31]. Can these lightning-induced radio pulses be responsible for the effects studied in this work? Before we answer this question we first recall [3] the basic features of the TA bursts interpreted as the AQN events, see item 1. Then, in item 2 we overview the main features of the radio emission which always accompanies the thunderstorm lightning events. After that in item 3 we explain the dramatic qualitative differences between the radio pulses associated with thunderstorm lightnings and the radio pulses studied in this work.

**1.** As we previously mentioned, lightnings are correlated with AQN events when an AQN hits the region of the thunderclouds and potentially may initiate lightnings serving as a trigger [3]. The mechanism is similar to the role of conventional CRs that are considered to be one of the dominant triggers of lightnings, see footnote 4 with related comments and references. This is the basic reason why the bursts being interpreted as random AQN events are nevertheless associated with the lightnings.

Further to this point, it is important to emphasize that in the AQN framework the direction of the electric field ${\mathscr {E}}$ characterized by (2) (also shown on Fig. 1) and the direction of the lightning current are not correlated. This is because the AQN event may serve as a trigger for the lightning strike. The trigger should not be confused with the initial stepped lightning leader which determines the direction of the current of the lightning being developed. In different words, the direction of the observed current of a lightning does not determine the sign of the fluctuating electric field ${\mathscr {E}}$ which itself defines the direction of the positrons to be recorded as TASD unusual events.

To rephrase it, an AQN plays the dual role when it propagates in the thunderclouds: it emits the very energetic positrons, and it also triggers the lightning, similar to conventional CR, see footnote 4 with relevant references. The former phenomenon is recorded as a TASD burst, while the latter one explains the observed correlation between the bursts and lightnings.

As a next remark: as we mentioned, all mysterious bursts occur at the very initial moment of the lightning flashes or even earlier than lightning. Some bursts are not related to the lightning flashes at all (but occur under the thunderclouds). In original interpretation [1] this feature is explained in terms of the initial stepped lightning leaders propagating to the ground on a time scale of ten or tens of milliseconds. In the AQN framework this feature is also automatically satisfied as the AQNs can serve as the triggers of lightning flashes, which may or may not initiate the lightnings. The particles which are recorded as TASD events are produced directly by AQN itself at this moment, not at the initial stage of the strike determined by the lightning leader.

**2.** Having discussed the basic features of the TASD events within AQN framework we now turn to our next item on radio emission features as a result of lightnings. The correlation between lightning and radio emission during thunderstorms is well known and well documented generic feature of the the lightning discharges, see [40] with large number of references on observations. In particular, the lightning discharges are characterized by a very large number of radio pulses which last in total for about 1 s. Each pulse is characterized by full width (0.2–0.3) $\upmu \mathrm{s}$ with electric field strength which could be as large as $|\mathbf {E}|\sim 10^3~\hbox {mV}/\hbox {m}$. Most of the pulses, though, show the strength of the electric field in the $|\mathbf {E}|\sim (100\text {--}200)~\hbox {mV}/\hbox {m}$ range. Another important feature of the radio emission: the gaps between pulses are in the range $(10--10^2) \, \upmu \mathrm{s}$. Therefore, total number of pulses could be very large $\gtrsim 10^3$ during a single lightning event. Finally, the typical frequency of the radiation is strongly peaked in few MHz bands, while it completely diminishes for $\nu \gtrsim 10~\hbox {MHz}$.

**3.** These features must be contrasted with the AQN-induced radio pulses studied in this work. While a typical duration of an individual pulse $\tau _{{\mathscr {E}}}$ and the strength of the corresponding electric field $|\mathbf {E}|$ assume similar orders of magnitude in both cases, some other key characteristics are dramatically different. It allows an easy discrimination between these two cases.

Indeed, the number of clustered radio pulses associated with the mysterious bursts must be very few (it ought to be 3+ corresponding to the number of clustered events in a single burst). It should be contrasted with $\sim 10^3$ in case of the lightning-induced radio pulses. The total durations of the radio emissions are also very different for these two cases.

The most important distinct feature which discriminates the different sources of the emission is that the frequency bands of the radiation are dramatically different in these two cases. The lightning-induced radio emission is strongly peaked in few MHz bands, while AQN-induced radio pulse is characterized by the flat spectrum with $\nu \lesssim 200~\hbox {MHz}$ according to (23). The basic reason for this dramatic difference is that the electric current responsible for the lightning is represented by the particles with $\gamma \sim 1$ while for the AQN-induced case the positrons are characterized by $\gamma \sim 20$. This difference is translated into dramatic modification of the frequency bands according to (23) though the typical time scale for an individual lightning-induced pulse $\sim (0.2\text {--}0.3) \,\upmu \mathrm{s}$ assumes the same order of magnitude as $\tau _{{\mathscr {E}}}\sim 0.3\,\upmu \mathrm{s}$.

Next, we want to mention that there are some dramatic differences between the radio pulses induced by conventional CR showers [41, 42] and the AQN-induced radio pulses. These differences have been discussed in details in [28]. Here we want to mention that these dramatic differences are related to very different structures of the showers. In case of conventional CR the radio emission is based on the picture when a CR air shower is characterized by the “pancake” of particles and its central axis. The density of the particles strongly depend on the distance to the central axis such that the spectral density of the radio pulse is highly sensitive to the width of the “pancake”, which becomes much thicker further away from the axis. It is very different from the AQN-induced radio signal as the notions of the shower axis and the “pancake” do not exist in our case such that the density of the particles is approximately the same irrespective of the distance to the central axis.

Additionally, conventional CRs are well studied even being heavily distorted by electric field in thundercloud, and in fact it is nowadays an efficient probe to study the electric field in thunderstorms by analysing the radio emission [43].^5^ The dramatic difference with the radio pulse studied in the present work is that the radio emission due to the CR is strongly correlated with the Earth’s magnetic field, in contrast with dominant ${\mathscr {E}}$-type emission studied in the present work. As a result the width of the corresponding signal is in nanosecond range, in contrast with the AQN induced pulse when the width is $\sim 0.3 \,\upmu \mathrm{s}$. Furthermore, the polarization pattern for the pulses related to CRs are dramatically different from the AQN-induced emission. Therefore, there should be no technical difficulty to distinguish an AQN-induced radio pulse from a conventional CR under the thunderstorm. The corresponding studies could support or refute our proposal.

There are many other distinct features between the AQN and conventional CR air showers. In particular, the AQNs mostly emit photons in X ray bands, such that a signal cannot be observed by a fluorescence detector which is designed to detect the visible and UV light.^6^

Next, we note that gamma rays can be produced by an avalanche of relativistic runaway electrons during initiation of lightning [46]. Bursts of gamma rays initiated in the Earth’s atmosphere, commonly referred to as terrestrial gamma ray flashes, can be generated in a lightning leader system [47, 48]. The terrestrial gamma ray flashes can trigger atmospheric photonuclear reactions that produce neutrons and positrons [49]. Evidently these gamma rays and their byproducts do not interfere with our proposed radio detection, as they are excessively more energetic and only appear at frequency band above EHz. Another important point here is that this powerful emission occurs in a latter stage of a lightning, while an AQN-induced event appears before or at the initial stage as it serves as a potential trigger of a lightning strike.

Based on these dramatic differences in frequencies and timings of the radio emissions we conclude that the signals due to the thunderstorm lightning events and conventional CR air showers can be easily discriminated from the AQN-induced radio pulses studied in this work. Therefore, we suggest to study the corresponding radio signal to support or refute our proposal to interpret the TA bursts as the AQN-induced events.

Lastly, one can assume that the source of the TASD bursts is entirely due to the complicated and not well understood physics of the lightning strikes (which may or may not be recorded by the system on the surface). In addition to many problems with explanation of the observed intensity, timing, clustering features, and geometry, there is an additional problem of the very low observed event rate (which is 10) in comparison with recorded $\sim 10^4$ number of lightnings in the same area. At the same time the estimated event rate [3] based on assumption that the observed TASD bursts is a result of the AQN-induced events is consistent with observations.

To conclude: we are not aware of any studies which could explain the observed intensity, timing, clustering features, and geometry of the signal in form of the mysterious bursts being produced during initial stage of the lightning as recorded, while proposal [3] naturally explains all these features within AQN framework.

## Conclusion

As we stated in the Introduction the main goal of the present work is to test the proposal [3] by searching for the radio signals in frequency band $\nu \in$ (0.5–200) MHz which must be synchronized with the TA bursts. Such test would unambiguously support or refute the proposal (interpreting TA bursts as the AQN events) as the radio signals due to the AQN annihilation events can be easily discriminated from conventional radio pulses which always accompany the thunderstorm. This is precisely the goal of this work: we want to eliminate all common objections which essentially state that a thunderstorm is very complicated system^7^ such that everything is possible, including TA bursts as a result of flashes. The proposal of the present work is to study the radio signals in frequency band $\nu \in$ (0.5–200) MHz. The corresponding results would unambiguously answer these rhetoric questions, see Sect. 8 with more comments on this.

Now we summarize the results of our studies. As we argued at the very end of previous Sect. 7 the ${\mathscr {E}}$-type radiation plays the dominant role in radio emission. The corresponding results have been summarized at the very end of Sect. 5 where it has been argued that the radio pulse can be recorded if proper instruments are designed and built at the TASD site. The pulse must be synchronized with TASD bursts. Such a signal cannot be confused with any other spurious and noise signals as a result of this synchronization. Therefore, observing (not observing) such synchronized signals can confirm, substantiate or refute our proposal. We further argued in Sect. 8 that the AQN-induced radio signals can be easily discriminated from the pulses generated by the thunderstorm lightning events.

One should comment here that the strength (26) of the amplitude $|\mathbf {E}|$ on the level $20~\hbox {mV}/\hbox {m}$ with the duration time of the radio pulse on the scale $\sim 0.3\, \upmu \hbox {s}$ could be recorded with existing technology as discussed in [40] in application to thunderstorm lightning events where wide-band radio interferometry (0.1–30 MHz) has been used to detect such short signals with amplitudes of electric field on the level $10^2~\hbox {mV}/\hbox {m}$, which is very close to what is required for the purposes of the present work. Therefore, the predicted radio signals (26) can be in principle measured if proper instruments are designed and built at the TASD site.

Furthermore, the short radio pulses can be, in principle, recorded by any (sufficiently sensitive) radio telescope outside of the TASD site as the corresponding radio pulses will be emitted in form of the clustering events, similar to TA-bursts. Such clusters of individual short radio pulses can be discriminated from any spurious signals representing the radio noise. It can be also discriminated from radio emission occurring as a result of the thunderstorm lightning events as argued in Sect. 8.

Our present proposal suggests that the TA bursts with very unusual features will be synchronized with radio pulses. If this synchronization is observed and the interpretation of TA bursts as the AQN annihilation events is confirmed by future studies it would be the *direct* (non-gravitational) evidence which reveals the nature of the DM, in contrast with large number of *indirect* hints mentioned in Sect. 2.

## Data Availability

This manuscript has no associated data or the data will not be deposited. [Authors’ comment: This manuscript has no associated data because the results are based on analytical computations produced in this work.]

## Footnotes

1 Note that various names have been used since the early development of the model, such that AQN might be referred as the QCD ball, quark nugget, compact composite objects (CCO), etc. in the past literature.

2 In conventional baryon-antibaryon annhilation events, pions and muons are cospiously produced and consequenly generate a signigicant number of neutrinos and antineutrinos with energy 20–50 MeV that meets the high sensitivity range of SuperK.

3 One should comment here that the actual trajectories of the positrons are not the straight lines due to the Earth’s magnetic field ${{\mathscr {B}}}\sim 0.5$ gauss as we discuss in Sect. 6. However, the main point here is that the emitted positrons are characterized by the same velocity and localization at the instant of exit. Therefore the presence of the magnetic field ${{\mathscr {B}}}$ does not spoil our estimates for the temporal and spatial spreads given by Eq. (5) which must hold irrespective of the presence of ${{\mathscr {B}}}$.

4 It is known that the presence of a strong electric field (2) is required to initiate a runaway breakdown avalanche. Another element which remains to be a matter of debates [30–36] is the nature of the seeded particles which play the role of a trigger to an avalanche. The proposal [3] suggests that AQNs may also play the role of a trigger along with conventional sources such as the CR [30, 35].

5 Another way to study the electric field under the thunderstorm is to study distortion of the muon’s propagation in the background of electric field [44].

6 In fact, it was precisely the argument presented in [45] why the all sky camera has not observed any signal from a powerful meteor-like event detected by the infra-sound dedicated instrument.

7 For example, the question on what initiates and triggers a runaway breakdown avalanche still remains a matter of debates, in spite of many years of studies, see also footnote 4.
